# Expanding the bandwidth of checkpoint inhibitors for cancer using epigenetic regulators

**DOI:** 10.1172/JCI188611

**Published:** 2025-03-17

**Authors:** Daniel J. Brat

The discovery, development, and application of checkpoint inhibitors (CPIs) have deservedly received scientific and medical recognition at the highest levels, with the idea of unleashing immune cells on malignant cells expressing foreign neoantigens capturing the attention and imagination of all (1, 2). Their use has been cemented in the contemporary care of patients with cancer (3). CPIs have been effective for patients with specific cancer types (e.g., melanoma and non-small cell lung cancer) as well as other forms of cancer that have high mutational burdens, presumably because these cancer types have greater levels of neoantigens that lead to greater immunogenicity toward the unchecked T cell response (so called “hot tumors”) (4). Therapeutic responses of other forms of cancer, especially those with low mutation burden (“cold tumors”), have been lower, and acquired resistance remains a serious challenge. Rather than writing off CPIs for cold tumors or those with acquired resistance, one line of thought has been to enhance therapeutic efficacy by modulating the tumor microenvironment. Poor responses and acquired resistance are often due to the suboptimal interaction of tumor cells with the immune system due to the coexpression of multiple checkpoint pathways, the exclusion or exhaustion of T cells, or the loss of HLA expression or presentation.

Head and neck squamous cell carcinomas (HNSCC) are a tumor type that could benefit greatly from enhanced response rates to CPI (5, 6). A subset of patients with advanced disease benefit from therapies targeting the PD-1:PD-L1 interaction, yet the number of unresponsive patients is far greater. In a recent investigation published in this issue of the *JCI*, Qin, Mattox, and colleagues addressed the shortcoming of CPI in advanced HNSCC by initiating a phase 1b clinical trial using a pharmacological epigenetic regulator, 5-azacytidine (5-aza), in combination with CPIs in patients with recurrent and/or metastatic HNSCC who had failed chemoradiation and had progressed on initial CPI therapy (7). The hypothesis was that 5-aza could enhance immune responses of CPIs through its effects on DNA methylation by transcriptionally reprogramming immune cells and tumor cells within the TME by reducing immunosuppression and upregulating HLA class 1 components and tumor antigens, respectively. The trial was small, with only 13 participants enrolled and only 8 providing on-treatment tissue biopsies for study. Nonetheless, the results obtained were highly encouraging by almost all endpoints measured. Following 5-aza/CPI treatment, the neutrophil-to-lymphocyte ratio trended lower by 50%, which is a clinically favorable sign; global methylation within the TME decreased in three-fourths of the participants, indicating a robust treatment effect; tumor immunogenicity improved based on the IFN-γ signature and PD-L1 expression in a subset of patients; reduced CD4^+^ T regulatory cells were consistently seen, and CD8^+^ cells were increased in a subset; genes related to antigen processing, processing, and antigenicity were increased, as were those related to immune pathway and tumor suppressor activation. While the trial was not intended to address clinical outcomes, the impact on disease progression and survival were noteworthy (7).

All the caveats of an initial report emanating from a small cohort phase 1 trial apply here. The therapy needs to be validated in larger, controlled cohorts, among patients with uniform disease types and profiles, where outcomes and biomarker studies are powered more appropriately. Nonetheless, Qin et al. (7) clearly indicates that epigenetic reprogramming occurred, shifting TME profiles in a favorable direction and suggesting future trials and practice could demonstrate a survival benefit.

## Version 1 03/17/2025

Electronic publication
